# Ion Channel Dysfunction and Therapeutic Targeting in Salivary Gland Disorders

**DOI:** 10.1111/odi.70419

**Published:** 2026-07-05

**Authors:** Tarek Mohamed Abd El‐Aziz, Shankara N. Varadarajan, Viktor Drel, Prakash Arullampalam, Mahmoud I. Shoulkamy, Ahmed Al‐Sabi, Brij B. Singh

**Affiliations:** ^1^ Center for Regenerative Sciences, School of Dentistry University of Texas Health Science Center San Antonio San Antonio Texas USA; ^2^ Zoology Department, Faculty of Science Minia University El‐Minia Egypt; ^3^ Department of Biology, Faculty of Education and Arts Sohar University Sohar Sultanate of Oman; ^4^ College of Integrative Studies Abdullah Al Salem University Khaldiya Kuwait

**Keywords:** ion channels, radiation injury, salivary glands, Sjögren's disease, therapeutic targeting, xerostomia

## Abstract

**Objective:**

Salivary gland hypofunction and xerostomia represent major clinical complications of radiation therapy, autoimmune disorders such as Sjögren's disease, and inherited epithelial ion transport defects. This review integrates current knowledge on ion channel dysfunction as a central mechanistic driver of salivary gland pathology and evaluates promising ion channel‐targeted therapeutic strategies.

**Methods:**

A comprehensive narrative review was performed using PubMed, Scopus, and Web of Science to identify experimental, translational, and clinical studies on ion channel regulation in salivary gland physiology and disease.

**Results:**

Salivary secretion depends on coordinated Ca^2+^ signaling and polarized ion transport in acinar and ductal cells. Across radiation injury, Sjögren's disease, and cystic fibrosis, disease‐specific disruptions of calcium entry, chloride conductance, and water channel trafficking converge on impaired fluid secretion through distinct but mechanistically overlapping pathways. These insights have driven targeted therapies, including muscarinic agonists, ANO1 and CFTR modulators, anti‐inflammatory strategies, and AAV2‐hAQP1 gene therapy, which shows sustained benefit in radiation‐induced xerostomia.

**Conclusions:**

Ion channels are central regulators of salivary gland physiology and promising therapeutic targets, and combining pharmacologic, gene, and regenerative approaches provides a precision strategy to restore salivary gland function.

**Abbreviations**
AAV2: Adeno‐associated virus serotype 2
ANO1: Anoctamin 1 (TMEM16A)
AQP1/AQP3/AQP5: Aquaporin 1/3/5
BK (KCa1.1): Large‐conductance calcium‐activated potassium channel
CF: Cystic fibrosis
CFTR: Cystic fibrosis transmembrane conductance regulator
DAMPs: Damage‐associated molecular patterns
EMT: Epithelial‐mesenchymal transition
ENaC: Epithelial sodium channel
ER: Endoplasmic reticulum
ERM: Ezrin‐radixin‐moesin
IK (KCa3.1): Intermediate‐conductance calcium‐activated potassium channel
IL‐1β: Interleukin‐1 beta
IP_3_R: Inositol 1,4,5‐trisphosphate receptor
JNK: c‐Jun N‐terminal kinase
LAMP3: Lysosome‐associated membrane protein 3
MCU: Mitochondrial calcium uniporter
NFAT: Nuclear factor of activated T‐cells
NKCC1: Na^+^‐K^+^‐2Cl^−^ cotransporter 1
NLRP3: NOD‐like receptor protein 3
NOX4: NADPH oxidase 4
ORAI1/ORAI2: Calcium release‐activated calcium channel proteins 1/2
P2X7R: P2X Purinoceptor 7 receptor
PARP1: Poly(ADP‐ribose) polymerase 1
PGE2: Prostaglandin E2
PIEZO1: Piezo‐type mechanosensitive ion channel component 1
RMAT: Regenerative Medicine Advanced Therapy
ROS: Reactive oxygen species
SjD: Sjögren's disease
SKF‐96365: Non‐selective SOCE/TRPC channel inhibitor
SOCE: Store‐operated calcium entry
STIM1/STIM2: Stromal interaction molecule 1/2
TGF‐β1: Transforming growth factor beta 1
TMEM16A: Transmembrane member 16A (Anoctamin 1)
TRPC1: Transient receptor potential canonical 1
TRPM2: Transient receptor potential melastatin 2
YM‐58483: Selective ORAI/CRAC channel inhibitor (also known as BTP2)

## 1 Introduction

Salivary gland hypofunction and xerostomia are prevalent clinical conditions that severely compromise oral health, nutrition, and quality of life. Saliva is essential not only for lubrication and digestion but also for antimicrobial defense, buffering of oral pH, and maintenance of mucosal integrity. Consequently, sustained reductions in salivary flow markedly increase the risk of dental caries, oral infections, dysphagia, and impaired speech (Humphrey and Williamson 2001; Dawes et al. 2015; Jensen and Vissink 2014). Xerostomia arises in diverse pathological conditions, including radiation therapy for head and neck cancer, Sjögren's disease (SjD), and inherited disorders of epithelial ion transport (Jensen and Vissink 2014; Pai et al. 2025; Fox 2005). Salivary secretion is driven by tightly coordinated ion transport processes within acinar and ductal cells. Acinar cells generate primary isotonic fluid through calcium‐dependent activation of chloride and potassium channels, while ductal cells subsequently modify their ionic composition to produce hypotonic final saliva (Abd El‐Aziz and Singh 2026). This highly regulated system depends on precise spatial and temporal control of calcium signaling, membrane conductances, and epithelial polarity. Increasing evidence indicates that disruption of these ion channel‐dependent processes represents a common mechanistic basis underlying salivary gland dysfunction across multiple diseases (Melvin et al. 2005; Ambudkar 2016; Petersen and Tepikin 2008).

Radiation‐induced xerostomia provides a prominent example of ion channel‐centered pathology. Exposure of salivary glands to ionizing radiation results in early oxidative stress, persistent disturbances in calcium homeostasis, mitochondrial dysfunction, and loss of secretory capacity, which often lead to extensive acinar cell loss (Jasmer et al. 2020; Coppes and Stokman 2011). TRPM2, which is activated by oxidative and metabolic stress, has been demonstrated as critical mediators linking radiation‐induced DNA damage to sustained calcium overload, cellular senescence, and irreversible glandular hypofunction (Yamamoto et al. 2008; Takahashi et al. 2011; Liu et al. 2013). These findings underscore that salivary failure after radiation is not only a consequence of cell death but reflects dysregulated stress signaling within surviving epithelial cells.

Autoimmune disorders such as SjD further illustrate the pathological impact of ion channel dysregulation. Notably, salivary glands in SjD often retain structural integrity while exhibiting profound secretory dysfunction. This dissociation reflects early defects in Ca^2+^ signaling pathways, including impaired IP_3_ receptor‐mediated Ca^2+^ release, reduced TRPC1‐dependent Ca^2+^ entry, and mislocalization of aquaporin‐5 (AQP5), which collectively disrupt epithelial fluid secretion prior to obvious immune‐mediated tissue destruction (Chivasso et al. 2023; Steinfeld and Delporte 2002; Ambudkar 2018). Similarly, in cystic fibrosis, impaired CFTR function disrupts ductal ion transport and saliva composition, while acinar fluid secretion is partially preserved through compensatory chloride conductances (Quinton 1999; Muñoz et al. 2024). Together, these conditions highlight how selective disruption of epithelial ion channel networks can separate glandular structure from function.

In addition to inherited and acquired defects in epithelial ion transport, ion channels have appeared as central regulators of stress signaling, inflammatory responses, and tissue remodeling within salivary glands. Mechanosensitive channels, volume‐regulated anion channels, and redox‐sensitive calcium‐permeable channels have been implicated in epithelial stress responses, inflammatory signaling, and regulation of regenerative capacity in salivary tissue (Coste et al. 2012; Qiu et al. 2014; Osei‐Owusu et al. 2018; Golnarnik et al. 2024).

This review examines how ion channel dysregulation drives salivary gland disease across three distinct but mechanistically interconnected etiologies. Sections 2 and 3 trace the molecular pathways from physiological ion transport to disease‐specific dysfunction, encompassing oxidative stress, inflammatory signaling, mechanotransduction, and epithelial remodeling, while Section 4 evaluates how these mechanistic insights are translating into targeted diagnostics, pharmacological interventions, gene therapy, and regenerative strategies.

## 2 Ion Channel Machinery in Salivary Gland Secretion

Salivary fluid secretion is a highly coordinated epithelial transport process that depends on the integrated function of multiple ion channels, transporters, and water channels distributed across polarized acinar and ductal cells (Figure 1).

**FIGURE 1:**
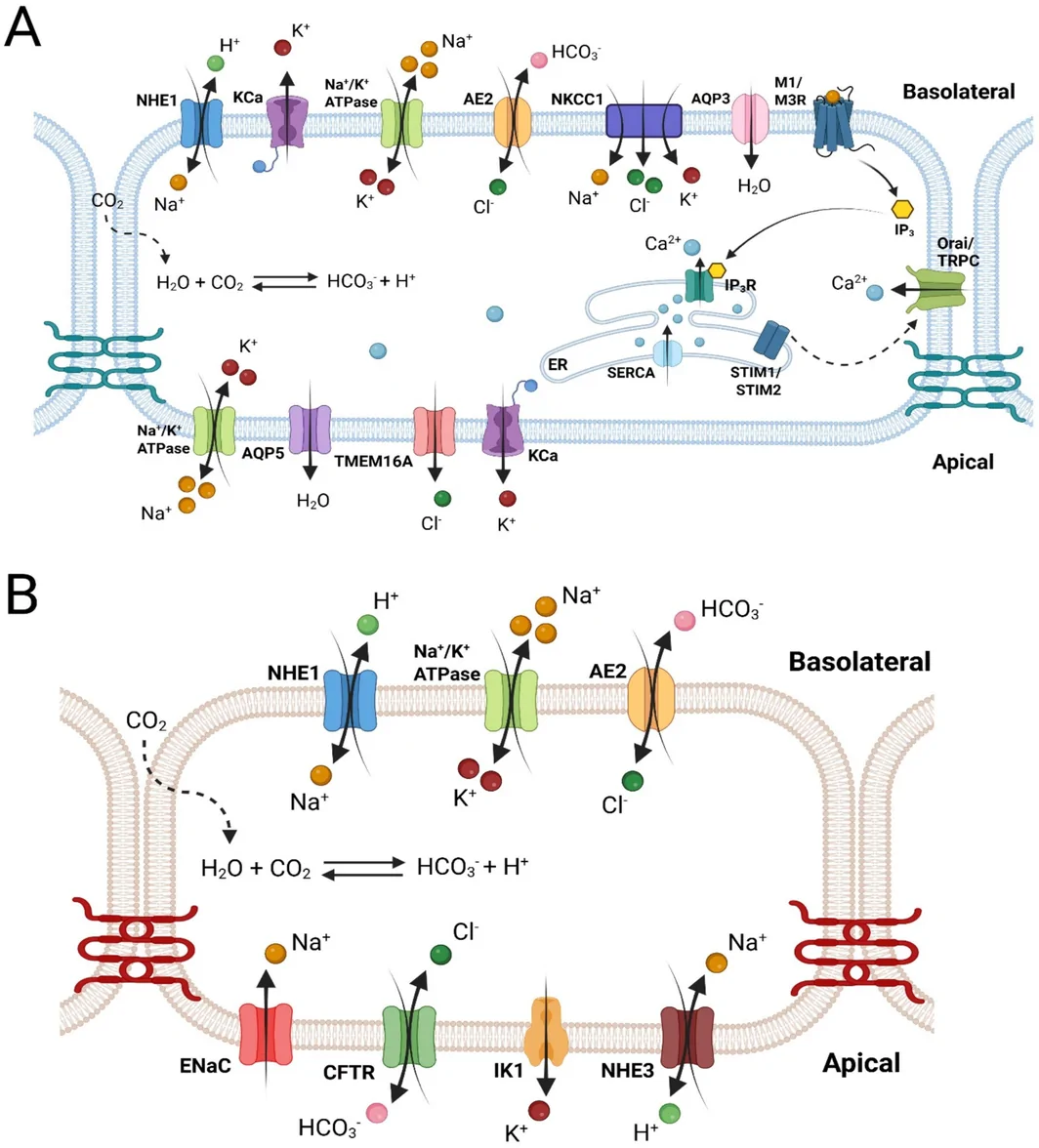
Ion channels and transporters salivary gland acinar and ductal epithelial cells. (A) Schematic representation of ion channels, transporters, and signaling pathways regulating fluid and electrolyte secretion in salivary gland acinar cells. (B) Overview of ion transport processes in ductal cells, illustrating Na^+^ reabsorption, Cl^−^/HCO_3_^−^ exchange, and electrolyte modification of primary saliva during ductal passage. AE2, anion exchanger 2; AQP3/5, aquaporin 3/5; CFTR, cystic fibrosis transmembrane conductance regulator; ENaC, epithelial sodium channel; ER, endoplasmic reticulum; IK1, intermediate‐conductance Ca^2+^‐activated K^+^ channel; IP_3_, inositol 1,4,5‐trisphosphate; IP_3_R, IP_3_ receptor; KCa, Ca^2+^‐activated K^+^ channel; M1/M3R, muscarinic acetylcholine receptor 1/3; Na^+^/K^+^‐ATPase, sodium–potassium ATPase; NHE1/3, Na^+^/H^+^ exchanger 1/3; NKCC1, Na^+^‐K^+^‐2Cl^−^ cotransporter 1; Orai, Ca^2+^‐permeable channels; SERCA, sarco/endoplasmic reticulum Ca^2+^‐ATPase; STIM1/2, stromal interaction molecule 1/2; TMEM16A, Ca^2+^‐activated Cl^−^ channel (ANO1).

### 2.1 Calcium Signaling as the Master Regulator of Secretion

Neurotransmitter‐stimulated increases in cytosolic calcium concentration ([Ca^2+^]ᵢ) represent the primary trigger for salivary fluid secretion (Melvin et al. 2005; Ambudkar 2014). Parasympathetic stimulation activates muscarinic M1/M3 receptors coupled to phospholipase C, generating IP_3_ that releases Ca^2+^ from endoplasmic reticulum stores via IP_3_ receptors (Ambudkar 2012, 2014). This initial Ca^2+^ release is spatially organized, originating in the apical region of acinar cells before propagating as a wave toward the basal pole (Ambudkar 2012; Hong et al. 2011). Sustained secretory responses require Ca^2+^ entry from the extracellular space through the store‐operated Ca^2+^ entry (SOCE) mechanism (Ambudkar 2014; Cheng et al. 2013). Depletion of endoplasmic reticulum Ca^2+^ stores activates STIM1, an ER‐resident Ca^2+^ sensor that oligomerizes and translocates to ER‐plasma membrane junctions, where it activates Orai1/TRPC1 channels (Cheng et al. 2013; Liu et al. 2005). TRPC1 channels contribute additional Ca^2+^ influx and appear functionally coupled to Orai1‐mediated entry (Liu et al. 2005; Ong et al. 2007). Genetic deletion of STIM in mice severely impairs sustained salivary secretion, demonstrating that SOCE is indispensable for physiological gland function (Son et al. 2024). The spatial and temporal characteristics of Ca^2+^ signals are crucial for the proper activation of downstream effectors.

### 2.2 Chloride Secretion: The Rate‐Limiting Step

Transepithelial chloride movement provides the primary osmotic driving force for fluid secretion in salivary acinar cells (Turner and Sugiya 2002; Romanenko et al. 2010). ANO1 (TMEM16A), a calcium‐activated chloride channel localized to the apical membrane, has been identified as the molecular correlation of this secretory conductance (Romanenko et al. 2010; Yang et al. 2008; Schroeder et al. 2008). ANO1 activation requires direct binding of Ca^2+^ to residues within the channel protein, with half‐maximal activation occurring at Ca^2+^ concentrations achieved during physiological stimulation (Yang et al. 2008; Pedemonte and Galietta 2014). Mice with targeted disruption of the Tmem16A gene exhibit severely impaired Ca^2+^‐dependent salivary secretion, with loss of the characteristic Ca^2+^‐activated chloride current in acinar cells (Romanenko et al. 2010; Schroeder et al. 2008; Ousingsawat et al. 2009). Importantly, ANO1 carries nearly all calcium‐activated chloride current in salivary gland epithelium, distinguishing this tissue from airways and intestine where other chloride channels contribute substantially (Namkung, Phuan, and Verkman 2011). This tissue specificity positions ANO1 as a particularly attractive therapeutic target for salivary gland disorders. ANO1 associates with ezrin‐radixin‐myosin (ERM) scaffolding proteins that link the channel to the actin cytoskeleton and anchor it at the apical membrane (Perez‐Cornejo et al. 2012). This interaction is functionally significant, as disruption of the ERM network reduces ANO1 surface expression and chloride conductance.

### 2.3 Water Transport Through Aquaporins

Water movement across salivary acinar cells occurs through water channels that provide high membrane permeability to osmotically driven water flux (Matsuzaki et al. 1999; Ishikawa et al. 2002). AQP5, localized predominantly to the apical membrane, is the principal water channel facilitating transcellular water transport into the acinar lumen (Matsuzaki et al. 1999; Ishikawa et al. 2002; Krane et al. 2001). AQP3 at the basolateral membrane provides the entry pathway for water from the interstitium (Ishikawa et al. 2002). The functional importance of AQP5 has been demonstrated in knockout mice, which exhibit decreased salivary flow (~60% reduction) and production of viscous, hypertonic saliva following pilocarpine stimulation (Krane et al. 2001; Ma et al. 1999). The elevated tonicity reflects unimpaired salt secretion across a relatively water‐impermeable epithelium, confirming that AQP5 is rate‐limiting for water movement (Ma et al. 1999). AQP5 distribution is dynamically regulated and requires proper epithelial polarity for appropriate apical localization (Steinfeld et al. 2001; Murdiastuti et al. 2002). Disrupted trafficking or mislocalization of AQP5 impairs water secretion even when total protein expression is preserved, a phenomenon with important implications, particularly in SjD (see Section 3.2.1) (Steinfeld et al. 2001; Beroukas et al. 2001).

### 2.4 Basolateral Ion Transport and Electrochemical Driving Force

Effective salivary fluid secretion requires maintenance of the electrochemical gradients that sustain apical chloride efflux (Romanenko et al. 2007; Begenisich et al. 2004). Calcium‐activated potassium channels, including KCa1.1 (BK) and KCa3.1 (IK), are activated during secretagogue stimulation and promote membrane hyperpolarization, thereby preserving the driving force for chloride movement through apical ANO1 channels (Romanenko et al. 2007; Begenisich et al. 2004; Nakamoto et al. 2008). Functional redundancy between these potassium channels ensures robust secretory capacity, although combined disruption significantly impairs fluid secretion. Simultaneously, intracellular chloride levels are replenished through the basolateral Na^+^‐K^+^‐2Cl^−^ cotransporter (NKCC1), which utilizes the sodium gradient generated by Na^+^/K^+^‐ATPase activity (Haas and Forbush 3rd 2000; Evans et al. 2000). This coordinated basolateral transport machinery maintains intracellular ionic balance and sustains continuous chloride secretion during prolonged stimulation. Together, potassium channel‐mediated hyperpolarization and NKCC1‐dependent chloride uptake form the essential basolateral framework supporting salivary fluid secretion.

### 2.5 Ductal Modification of Primary Saliva

As isotonic primary saliva pass through the ductal system, its composition is substantially modified through electrolyte reabsorption and secretion (Varga 2015). Ductal epithelia reabsorb Na^+^ and Cl^−^ while secreting K^+^ and HCO_3_^−^, converting isotonic primary saliva into the hypotonic final secretion characteristic of healthy salivary function (Patterson et al. 2012). ENaC, located on the apical membrane of duct cells, mediates electrogenic sodium reabsorption (Catalán et al. 2010). ENaC consists of three homologous subunits (α, β, γ) and provides the rate‐limiting step for ductal sodium uptake (Catalán et al. 2010). CFTR, expressed apically in duct cells, facilitates chloride reabsorption and bicarbonate secretion (Catalán et al. 2010; Lee et al. 2012). CFTR function appears coupled to ENaC activity, with pharmacological blockade of either channel impairing both sodium and chloride handling (Catalán et al. 2010). Importantly, the ductal epithelium has low water permeability, permitting electrolyte reabsorption without equivalent water movement (Martinez 1987). This functional property enables the generation of hypotonic final saliva, which typically has Na^+^ and Cl^−^ concentrations well below plasma levels at normal flow rates.

## 3 Pathophysiological Impact of Ion Channel Dysfunction in Salivary Gland Disorders

Ion channel dysfunction in salivary glands impairs fluid and protein secretion, leading to xerostomia, increased susceptibility to oral infections, and diminished quality of life (Table 1) (Dirix et al. 2006; Jensen et al. 2010). Understanding the molecular mechanisms underlying these disorders is critical for developing targeted therapeutic interventions.

**TABLE 1:** Ion channel expression and functional changes in salivary gland disorders.

Disease	Key ion channels	Primary pathogenic mechanism	Key findings	References
Radiation‐induced xerostomia	TRPM2, STIM1, AQP5	Radiation‐induced oxidative stress activates TRPM2, triggering mitochondrial Ca^2+^ overload. This activates caspase‐3, which cleaves STIM1, causing sustained SOCE failure. AQP5 is downregulated through p53‐dependent pathways and NOX4‐mediated oxidative damage	TRPM2^−^/^−^ mice show preserved salivary function post‐irradiation; adenoviral STIM1 expression restores SOCE and fluid secretion; AQP5 expression reduced 40%–60% by day 30 post‐irradiation	(I. S. Ambudkar 2016; Liu et al. 2013; Ren et al. 2022; D'Agostino et al. 2020; Meyer et al. 2019; Li et al. 2006; Chivasso et al. 2021; Takakura et al. 2007; Liu et al. 2017; Cheng et al. 2011)
Sjögren's disease	AQP5, TRPC1, IP3R	AQP5 redistributes from apical to basolateral membranes due to cytokine‐induced disruption of tight junctions and ezrin scaffolding. TRPC1 downregulation (via IL‐17/IκB‐ζ pathway) reduces SOCE, causing ER stress and alarmin release that triggers immune infiltration. IP3R2/3 loss impairs initial Ca^2+^ release	AQP5 mislocalization occurs independently of lymphocytic infiltration; TRPC1 loss precedes immune cell infiltration; paquinimod (alarmin inhibitor) reverses SjD phenotype in IL14α mice; IL‐17 neutralization restores TRPC1 and saliva flow	(Chivasso et al. 2023; Steinfeld et al. 2001; Soyfoo et al. 2018; Konttinen et al. 2005; Hou et al. 2024; Fu et al. 2024; Nakamura et al. 2022; Teos et al. 2015; Pauley et al. 2011; Sun et al. 2023; Zhou et al. 2024; Xiao et al. 2021)
Cystic fibrosis	CFTR, ENaC, ANO1	CFTR mutations impair ductal Cl^−^ reabsorption and HCO_3_^−^ secretion. Loss of CFTR‐ENaC functional coupling reduces Na^+^ reabsorption. ANO1/TMEM16A provides a CFTR‐independent Ca^2+^‐activated Cl^−^ pathway that maintains acinar secretion and preserves gland morphology	CF patients show elevated salivary Na^+^ and Cl^−^; salivary glands retain relatively preserved morphology unlike pancreas; TMEM16A activators increase submucosal gland secretion independent of CFTR	(Romanenko et al. 2010; Catalán et al. 2010; Wine 1999; Quinton 2008; Zinn et al. 2015; Crawford et al. 1991; Roomans 2010; Noël et al. 2008; Quinton 2007; Namkung, Yao, et al. 2011; Benedetto et al. 2017)

### 3.1 Radiation Therapy: Ion Channel Dysregulation and Mechanisms of Xerostomia

Radiotherapy for head and neck cancers disrupts acinar fluid secretion, impairs ductal electrolyte transport, and alters epithelial homeostasis, leading to persistent hyposalivation and xerostomia (Blitzer et al. 2025; Eisbruch et al. 2001). Chronic xerostomia develops in approximately 50%–70% of patients receiving conventional head and neck radiotherapy, making it one of the most prevalent and debilitating late complications of cancer treatment (Blitzer et al. 2025; Eisbruch et al. 2001; Bhide et al. 2009). Although modern radiation delivery strategies have reduced the incidence of severe xerostomia to approximately 30%, a substantial proportion of patients continue to experience sustained salivary hypofunction. In contrast to early hypotheses that attributed radiation‐induced hyposalivation primarily to extensive acinar cell apoptosis, recent evidence indicates that cellular senescence, rather than apoptosis, drives chronic salivary gland dysfunction (Marmary et al. 2016; Gunning and Limesand 2024). At clinically relevant radiation exposures, typically delivered as fractionated doses totaling 50–70 Gy over 5–7 weeks in patients, experimental models often employ single‐dose irradiation ranging from 5 to 15 Gy to recapitulate key aspects of glandular injury. Under these conditions, apoptosis markers such as caspase‐3 activation and TUNEL staining remain relatively low, whereas histological analyses reveal progressive accumulation of senescent acinar cells characterized by enlarged, hyperchromatic nuclei (Marmary et al. 2016). These senescent cells retain AQP5 expression, confirming their acinar origin, yet exhibit profound secretory dysfunction (Marmary et al. 2016). The molecular basis of radiation‐induced xerostomia centers on ion channel and water channel dysregulation.

#### 3.1.1 Radiation‐Induced Aquaporin‐5 Dysregulation

AQP5, the primary apical water channel in acinar cells, is significantly altered by irradiation (Ren et al. 2022; D'Agostino et al. 2020; Meyer et al. 2019). Immunohistochemical studies demonstrated decreased AQP5 protein levels and abnormal subcellular localization in irradiated glands (D'Agostino et al. 2020; Meyer et al. 2019). Li et al. demonstrated that AQP5 mRNA expression was reduced by 37% on day 3 and 51% on day 30 post‐irradiation in rat submandibular glands, with corresponding decreases in protein levels of 40% and 60%, respectively (Li et al. 2006). This AQP5 dysfunction occurs through multiple mechanisms: (1) radiation‐induced oxidative stress mediated by NADPH oxidase 4 (NOX4) generates reactive oxygen species that damage AQP5 protein structure (Ren et al. 2022); (2) transcriptional downregulation of AQP5 expression via p53‐dependent pathways (Ren et al. 2022); (3) disrupted trafficking and membrane insertion of AQP5 resulting from cytoskeletal damage and loss of ezrin scaffolding proteins (Meyer et al. 2019; Chivasso et al. 2021). Takakura et al. demonstrated that pre‐treatment with the muscarinic receptor agonist cevimeline prevented radiation‐induced decreases in AQP5 expression and partially preserved saliva flow rates up to day 28 post‐irradiation, suggesting that maintaining AQP5 levels represents a viable radioprotective strategy (Takakura et al. 2007).

#### 3.1.2 TRPM2‐Mediated Calcium Overload and STIM1 Cleavage Following Irradiation

Radiation‐induced salivary gland dysfunction involves activation of the transient receptor potential melastatin 2 (TRPM2) channel (Figure 2). TRPM2 is a calcium‐permeable cation channel stimulated by oxidative stress and poly(ADP‐ribose) polymerase 1 (PARP1)‐mediated DNA damage responses (Liu et al. 2013, 2017). Following irradiation, TRPM2 activation triggers mitochondrial calcium overload via the mitochondrial calcium uniporter (MCU), resulting in increased mitochondrial ROS production, loss of membrane potential, and activation of caspase‐3 (Liu et al. 2017). Activated caspase‐3 subsequently cleaves STIM1, the ER‐resident Ca^2+^ sensor required for store‐operated Ca^2+^ entry (SOCE; see Section 2.1), thereby abolishing sustained Ca^2+^ influx through Orai1/TRPC1 channels (Liu et al. 2013, 2017). Loss of SOCE disrupts the prolonged Ca^2+^ signaling necessary for fluid secretion, establishing STIM1 degradation as a critical rate‐limiting step in radiation‐induced salivary gland dysfunction (Liu et al. 2017). Genetic deletion of TRPM2 in mice provides substantial protection against radiation‐induced salivary gland dysfunction, with TRPM2^−/−^ animals showing preserved salivary function at 10–30 days post‐irradiation (Liu et al. 2013, 2017). Similarly, pharmacological inhibition of TRPM2, PARP1, or caspase‐3 prevents radiation‐induced STIM1 loss and preserves SOCE function (Liu et al. 2017). Remarkably, adenovirus‐mediated expression of exogenous STIM1 in irradiated mouse salivary glands restores both SOCE and fluid secretion, demonstrating that STIM1 loss is a critical rate‐limiting step in radiation‐induced secretory failure (Liu et al. 2017).

**FIGURE 2:**
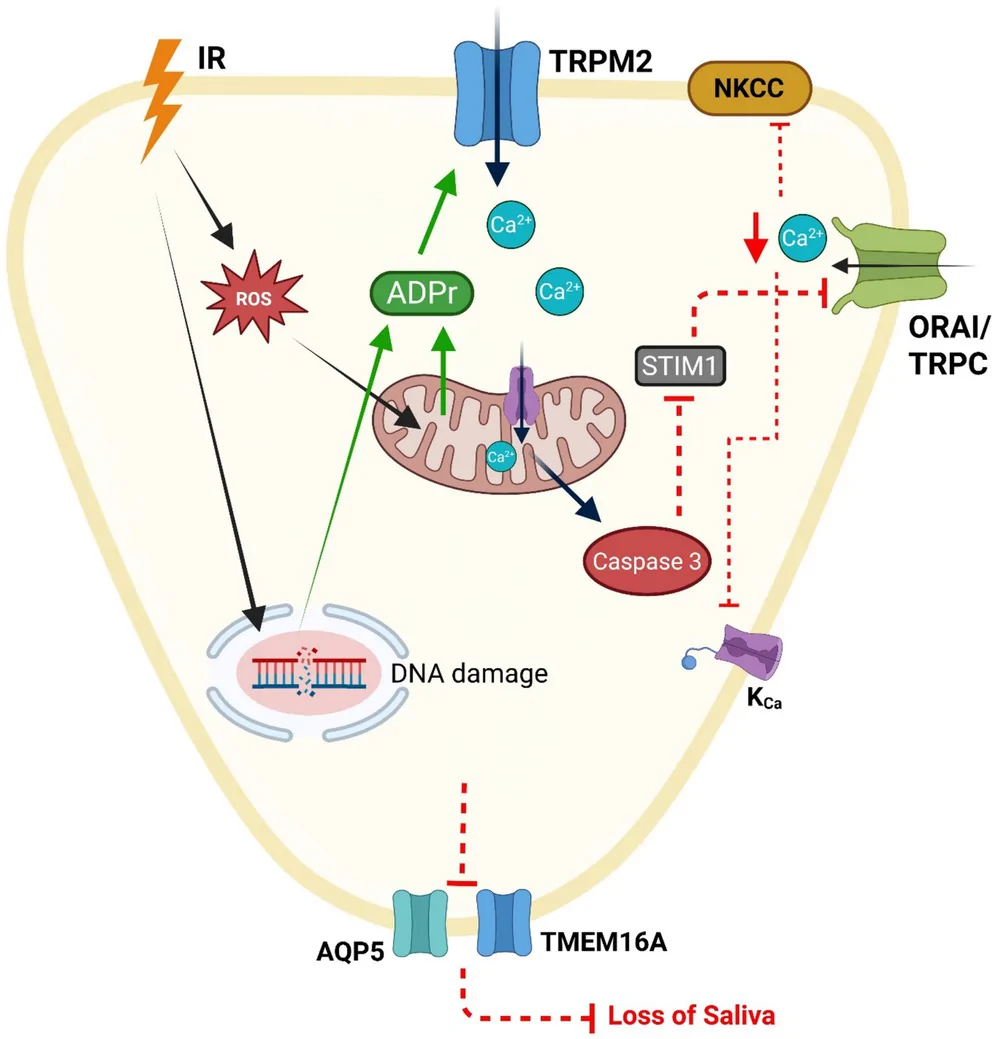
Radiation‐induced Ca^2+^ dysregulation and secretory failure in salivary acinar cells. Ionizing radiation (IR) induces reactive oxygen species (ROS) generation and DNA damage, leading to activation of poly(ADP‐ribose) polymerase and accumulation of ADP‐ribose (ADPr). ADPr activates the TRPM2 channel, promoting Ca^2+^ influx and mitochondrial Ca^2+^ loading. Excess mitochondrial Ca^2+^ contributes to mitochondrial dysfunction and caspase‐3 activation, resulting in loss of STIM1. Consequently, radiation impairs store‐operated Ca^2+^ entry through disrupted STIM1–ORAI/TRPC signaling and attenuates NKCC‐mediated Cl^−^ accumulation and Ca^2+^‐activated K^+^ channel (KCa) activity. Loss of coordinated Ca^2+^ signaling suppresses TMEM16A function and AQP5‐dependent water transport, ultimately leading to reduced saliva secretion.

#### 3.1.3 ORAI2‐Driven Fibrotic Remodeling After Radiation

ORAI2, a member of the ORAI family of calcium release‐activated calcium channels, has recently been identified as an important contributor to early‐stage post‐irradiation fibrosis in salivary glands (Li et al. 2025). RNA sequencing and bioinformatic analysis of mouse submandibular glands following 15 Gy irradiation revealed activation of calcium channel signaling pathways in both human patients and irradiated mice (Li et al. 2025). Mechanistic studies identified a novel ORAI2/JNK/NFAT1 signaling axis that drives TGF‐β1‐mediated fibrogenesis following irradiation (Li et al. 2025). Pharmacological inhibition of SOCE signaling using SKF‐96365, a non‐selective inhibitor of SOCE and TRPC channels, and YM‐58483 (also known as BTP2), a selective inhibitor of ORAI/CRAC channels, effectively blocked fibrosis at 30 days post‐irradiation in an ORAI2‐dependent manner (Li et al. 2025). Furthermore, pharmacological inhibition of NFAT1 using the calcineurin‐NFAT pathway inhibitor INCA‐6 significantly mitigated radiation‐induced fibrosis and restored saliva flow to 84.6% of normal levels, with no detectable side effects. These findings expand understanding of calcium channel involvement in radiation‐induced dysfunction beyond the acute phase, demonstrating that ORAI2‐mediated calcium signaling contributes to the chronic fibrotic remodeling that characterizes late‐stage radiation damage. Targeting the ORAI2/JNK/NFAT1 axis represents a promising therapeutic strategy to relieve radiation‐induced hyposalivation.

#### 3.1.4 P2X7 Receptor Activation and Radiation‐Induced Inflammatory Signaling

The P2X7 receptor (P2X7R) is a ligand‐gated ion channel activated by extracellular ATP released from damaged cells as damage‐associated molecular patterns (DAMPs). P2X7R activation initiates inflammatory responses and has been directly implicated in radiation‐induced salivary gland dysfunction (Jasmer et al. 2020; Gilman et al. 2019). Gilman et al. demonstrated dose‐dependent increases in γ‐radiation‐induced ATP release from primary parotid gland cells of wild‐type but not P2X7R^−^/^−^ mice (Gilman et al. 2019). Following 2–10 Gy irradiation, ATP is released from parotid cells immediately post‐exposure and activates P2X7R, leading to prostaglandin E2 (PGE2) release within 24–72 h (Gilman et al. 2019). Mice deficient in P2X7R showed reduced levels of both extracellular ATP and PGE2 release, which correlated with improved saliva flow at days 3–30 post‐irradiation (Gilman et al. 2019). Importantly, P2X7R‐dependent pathways did not impact cell death induction in parotid glands post‐irradiation, suggesting that P2X7R contributes to dysfunction through inflammatory signaling rather than apoptosis (Gilman et al. 2019). Through activation of the NLRP3 inflammasome, P2X7Rs promote ROS production, IL‐1β maturation and release, and subsequent inflammatory cascades (Jasmer et al. 2020). Activation of P2X7R also causes increased cytoplasmic calcium concentration and downstream signaling processes, including ERK1/2 signaling, gene expression changes, and further inflammatory amplification (Jasmer et al. 2020). Beyond radiation injury, P2X7R‐mediated inflammasome activation also plays a prominent role in autoimmune salivary gland pathology, as discussed in Section 3.2.3.

#### 3.1.5 PIEZO1 As a Radiation Biomarker and Mechanistic Contributor

PIEZO1, a mechanosensitive ion channel first discovered in 2010, has recently emerged as a novel predictive biomarker and potential pathogenic contributor in radiation‐induced xerostomia (Nguyen et al. 2024). PIEZO1 is significantly upregulated at both gene and protein levels during radiation‐induced salivary gland hypofunction. In a mouse xerostomia model receiving 15 Gy X‐ray irradiation to the head and neck, PIEZO1 upregulation was temporally associated with increases in inflammatory markers (F4/80) and fibrotic markers including fibronectin and collagen fiber accumulation (Nguyen et al. 2024). This temporal correlation suggests that PIEZO1 activation may contribute to the inflammatory and fibrotic processes underlying chronic radiation‐induced damage. PIEZO1 activation increases Ca^2+^ influx, which engages the same NLRP3 inflammasome pathway described in Section 3.1.4, thereby contributing to inflammation (Nguyen et al. 2024). In other irradiated tissues, PIEZO1 has been shown to contribute to radiation‐induced ferroptosis, an iron‐dependent form of regulated cell death characterized by lipid peroxidation, via Ca^2+^/calpain/VE‐cadherin signaling and to promote epithelial‐mesenchymal transition (EMT) through crosstalk with TGF‐β signaling (Guo et al. 2021; Huang et al. 2021). These findings suggest that PIEZO1 upregulation in salivary gland tissue may provide a novel predictive marker for radiation‐induced xerostomia, enabling earlier identification of patients at risk for developing chronic dysfunction (Nguyen et al. 2024). Furthermore, pharmacological modulation of PIEZO1 using antagonists such as GsMTx4, which has shown efficacy in reducing radiation‐induced damage in other tissues, represents a potential therapeutic avenue warranting further investigation in salivary glands.

### 3.2 Ion Channel Dysfunction in Sjögren's Disease

Primary SjD is a chronic, immune‐mediated autoimmune disease marked by lymphocytic infiltration and progressive dysfunction of exocrine glands. Inflammation of the salivary and lacrimal glands leads to hallmark clinical features, including dry mouth (xerostomia) and dry eyes (keratoconjunctivitis sicca) (Qin et al. 2015; Mavragani and Moutsopoulos 2014; Mariette and Criswell 2018). Affecting millions worldwide, SjD imposes a substantial burden on patients' quality of life. Current therapies are largely symptomatic and fail to address the underlying immunopathology, underscoring an urgent need for targeted and disease‐modifying interventions. Given the central role of ion channels in fluid secretion, emerging research is increasingly focused on targeting ion channel dysfunction as a promising avenue for restoring salivary gland function in SjD.

#### 3.2.1 Aquaporin‐5 Dysregulation as a Cardinal Pathogenic Mechanism

AQP5 dysfunction represents a central pathophysiological feature distinguishing SjD from other causes of salivary hypofunction (Chivasso et al. 2023; Steinfeld et al. 2001; Soyfoo et al. 2018). As described in Section 2.3, AQP5 is normally concentrated at the apical membrane of acinar cells (Steinfeld et al. 2001; Konttinen et al. 2005). Immunohistochemical analysis of salivary gland biopsies from SjD patients shows striking redistribution of AQP5 from apical to basolateral membranes, effectively inverting its normal polarization (Steinfeld et al. 2001; Konttinen et al. 2005). This mislocalization occurs independently of the degree of lymphocytic infiltration, suggesting that AQP5 aberration precedes or occurs parallel to immune‐mediated destruction (Chivasso et al. 2023; Hou et al. 2024). Multiple mechanisms contribute to AQP5 dysregulation in SjD (Chivasso et al. 2023; Fu et al. 2024): (1) Inflammatory cytokines, particularly TNF‐α and IFN‐γ, downregulate AQP5 transcription through NF‐κB and CREB signaling pathways (Fu et al. 2024); (2) Disruption of tight junction proteins (ZO‐1, occludin, claudin‐1) impairs epithelial cell polarity required for proper AQP5 trafficking (Hou et al. 2024; Fu et al. 2024); (3) Altered ezrin localization disrupts the scaffolding required for AQP5 membrane anchoring—ezrin normally links AQP5 to the actin cytoskeleton, and its mislocalization causes concurrent AQP5 redistribution (Hou et al. 2024; Fu et al. 2024); (4) Autoantibodies against AQP5 detected in sera from SjD patients may directly block channel function or trigger antibody‐mediated internalization (Beroukas et al. 2001; Fu et al. 2024); (5) LAMP3 (lysosome‐associated membrane protein 3) overexpression in SjD promotes endolysosomal degradation of AQP5 and other membrane proteins including NKCC1, compounding secretory dysfunction (Nakamura et al. 2022).

#### 3.2.2 Calcium Signaling Defects and Ion Channel Dysregulation

SjD involves broader disruptions of Ca^2+^ signaling machinery essential for fluid secretion (Figure 3). Several ion channels and calcium‐handling proteins have been directly implicated in SjD pathophysiology through experimental studies in human patient samples and mouse models of the disease.

**FIGURE 3:**
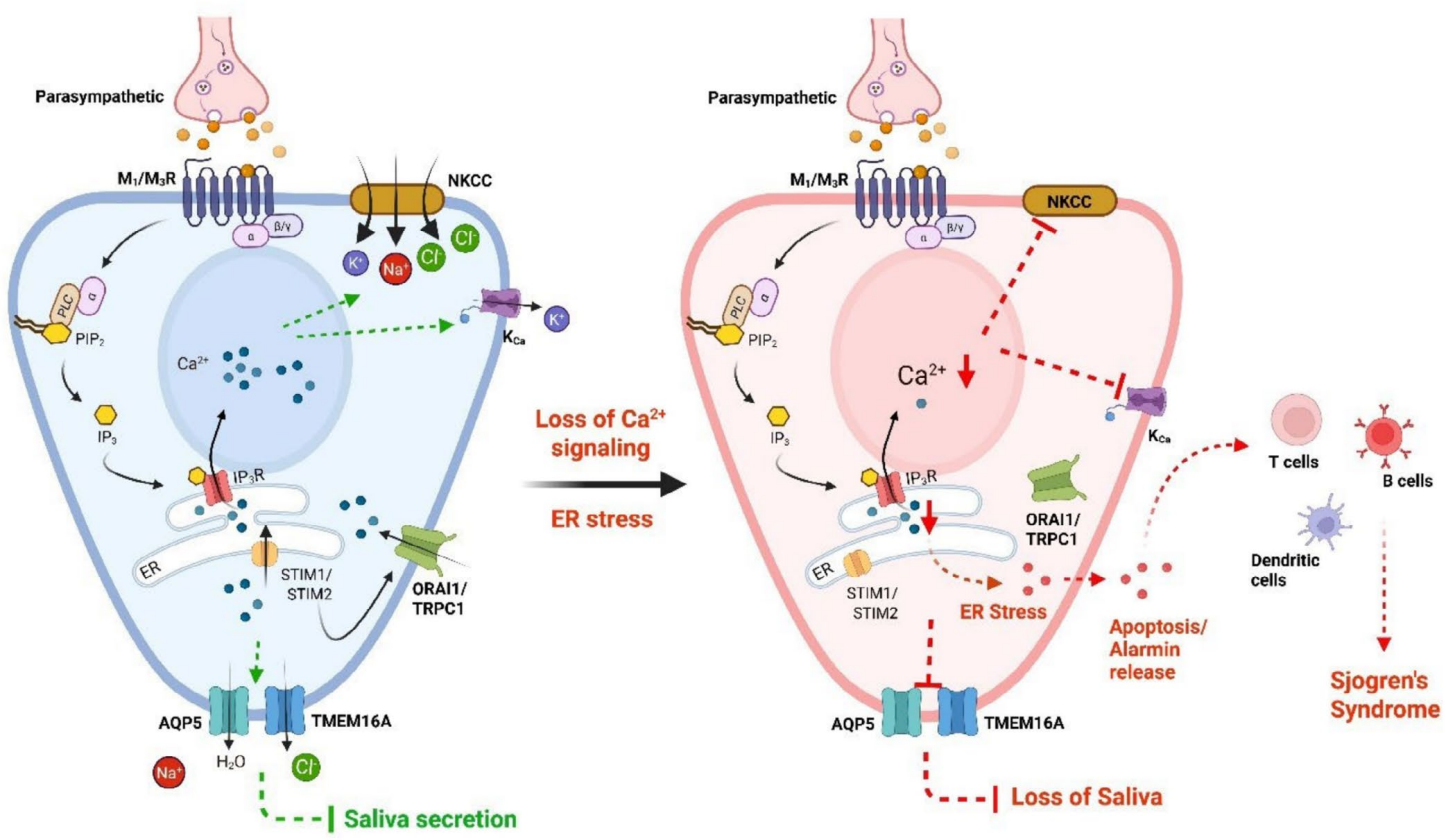
Impaired Ca^2+^ signaling as a central mechanism of salivary gland dysfunction in SjD. Left: Under physiological conditions, parasympathetic stimulation activates M_1_/M_3_ muscarinic receptors, initiating PLC‐IP_3_ signaling and Ca^2+^ release from the ER via IP_3_ receptors. ER Ca^2+^ depletion recruits STIM1/STIM2 and activates ORAI1/TRPC1 channels to sustain cytosolic Ca^2+^ elevations. Increased intracellular Ca^2+^ activates Ca^2+^‐dependent K^+^ channels (KCa) and TMEM16A, while NKCC maintains intracellular Cl^−^ loading. Coordinated Cl^−^ efflux and apical AQP5‐mediated water transport drive saliva secretion. Right: In SjD, impaired Ca^2+^ signaling disrupts store‐operated Ca^2+^ entry, ion channel coordination, and AQP5‐dependent water transport, leading to ER stress, apoptosis, and alarmin release. These events promote immune cell recruitment and progressive salivary gland dysfunction, resulting in reduced saliva secretion.

##### 3.2.2.1 IP3 Receptor Deficit

IP3Rs are critical calcium channels located in the endoplasmic reticulum that mediate the initial Ca^2+^ release required for salivary secretion. Teos et al. demonstrated that IP3R2 and IP3R3 are significantly reduced in morphologically intact acinar regions of minor salivary gland biopsies from SjD patients, even in individuals with mild lymphocytic infiltration (Teos et al. 2015). Critically, this deficit occurred without corresponding reductions in AQP5 or STIM1 expression, indicating that IP3R loss represents a distinct early pathogenic event (Teos et al. 2015). Functional calcium imaging studies confirmed that carbachol‐stimulated intracellular Ca^2+^ release was markedly attenuated in SjD acini compared to healthy controls, correlating with the observed IP3R reduction (Teos et al. 2015). Similar decreases in IP3R expression and Ca^2+^ signaling were detected in acinar cells from IL14α transgenic mice, a well‐characterized model of SjD (Teos et al. 2015). Treatment of healthy human salivary gland tissue with lymphotoxin‐α (LTα), a cytokine linked to disease progression in SjD, reduced Ca^2+^ signaling, suggesting that inflammatory cytokines may directly drive IP3R downregulation (Teos et al. 2015). The microRNA miR‐1248 has been implicated as a mechanistic link between autoimmune inflammation and IP3R deficit (Pauley et al. 2011). This microRNA exhibits dual functions: inhibiting IP3R translation while simultaneously inducing interferon production, thereby suppressing Ca^2+^ signaling and stimulating IFN signaling concurrently (Pauley et al. 2011).

##### 3.2.2.2 TRPC1 Downregulation and the Alarmin Pathway

TRPC1 is a major calcium entry channel that contributes to SOCE in salivary gland acinar cells. Sun et al. provided compelling evidence that TRPC1‐mediated Ca^2+^ signaling loss is an early pathogenic event in SjD that precedes immune cell infiltration (Sun et al. 2023). In the IL14α transgenic mouse model of SjD, TRPC1 protein and mRNA expression decreased progressively with age, correlating with declining salivary function (Sun et al. 2023). Importantly, loss of Ca^2+^ signaling preceded both decreased saliva secretion and immune cell infiltration, suggesting that channel dysfunction represents an initiating event rather than a consequence of autoimmune damage (Sun et al. 2023). TRPC1^−/−^ mice exhibited increased immune infiltration and abnormal immune cell presence in salivary glands, recapitulating features of SjD (Sun et al. 2023). Mechanistically, decreased TRPC1‐mediated Ca^2+^ entry leads to ER stress and acinar cell loss, which promotes the release of alarmins (damage‐associated molecular patterns) essential for immune cell infiltration and release of pro‐inflammatory cytokines (Sun et al. 2023). Human SjD patient samples similarly showed decreased TRPC1 expression with altered (diffuse) subcellular localization compared to the normal basolateral distribution, along with increased acinar cell death. Treatment of IL14α transgenic mice with paquinimod, an alarmin release inhibitor, restored Ca^2+^ homeostasis, inhibited alarmin release, decreased immune cell infiltration, and restored salivary gland function, thereby reversing the SjD phenotype (Sun et al. 2023). These findings establish TRPC1 as a central mediator of SjD pathophysiology and identify alarmin blockade as a potential therapeutic strategy.

Alarmins, also known as endogenous danger signals, are constitutively expressed intracellular molecules that are released upon cell damage or stress. In the context of SjD, the distinction between their roles in tissue protection versus recovery is critical: alarmin release from stressed acinar cells initially serves as a protective mechanism by signaling tissue damage and recruiting immune surveillance; however, sustained alarmin signaling due to ongoing TRPC1‐mediated calcium dysregulation shifts this response toward chronic inflammation and progressive glandular destruction rather than repair (Zhou et al. 2024). Ferroptosis (see Section 3.1.5) has also been implicated in SjD pathogenesis. CD4 T cell‐secreted IFN‐γ induces ferroptosis in salivary gland epithelial cells via the JAK/STAT1 pathway, leading to acinar cell loss distinct from apoptotic mechanisms (Zhou et al. 2024). While alarmin release represents an early, potentially reversible stage of cellular stress that may be amenable to therapeutic intervention (as demonstrated by paquinimod treatment), ferroptosis represents an irreversible endpoint of oxidative damage. This distinction has important therapeutic implications: targeting alarmin pathways may protect glandular function by preventing the transition from reversible stress to irreversible cell death, whereas anti‐ferroptotic strategies may promote recovery by preserving remaining acinar cells once damage has begun.

##### 3.2.2.3 STIM1/STIM2 and Immune Tolerance

SOCE mediated by STIM1, STIM2, Orai1, and TRPC channels is impaired in SjD (Cheng et al. 2012; Rosado et al. 2015). Early genetic studies demonstrated that T cell‐specific deletion of STIM1 and STIM2 in mice recapitulates key features of SjD, including exocrine gland dysfunction and autoantibody production, establishing SOCE components as critical regulators of immune tolerance in exocrine tissues (Cheng et al. 2012). Mice lacking *Stim1* and *Stim2* specifically in salivary gland epithelial cells (*Stim1/2* ^K14Cre(+)^) exhibited hyposalivation due to impaired SOCE and consequent loss of ANO1 activation; however, these mice notably showed no evidence of lymphocytic infiltration or autoantibodies characteristic of SjD, even with aging (Son et al. 2024). Furthermore, salivary gland biopsies from Sjögren's patients with high focus scores showed elevated rather than decreased STIM1 expression, accompanied by increased TLR7/8 expression (Son et al. 2024).

#### 3.2.3 P2X7 Receptor and Inflammasome Activation

In addition to its role in radiation‐induced inflammatory signaling (Section 3.1.4), P2X7R has emerged as a key mediator of salivary gland inflammation in SjD. The receptor is expressed on both acinar and ductal cells of submandibular and other salivary glands (Woods et al. 2012; Khalafalla et al. 2017). Woods et al. demonstrated that P2X7R activation with ATP or the high‐affinity agonist BzATP induces membrane blebbing, enhances caspase activity, and stimulates the cleavage and release of α‐fodrin in salivary gland cells (Woods et al. 2012). α‐Fodrin is a cytoskeletal protein thought to act as an autoantigen in SjD development, and its P2X7R‐mediated release may contribute to autoimmune targeting of salivary glands (Woods et al. 2012). In vivo administration of BzATP to ligated submandibular gland excretory duct enhanced immune cell infiltration and initiated apoptosis of salivary epithelial cells in wild type but not P2X7R^−/−^ mice. P2X7R activation in salivary gland cells engages the NLRP3 inflammasome–IL‐1β axis described in Section 3.1.4, with additional mechanistic requirements specific to this tissue context, including transmembrane Na^+^/K^+^ flux, heat shock protein 90 (HSP90) activation, and mitochondrial reactive oxygen species generation (Khalafalla et al. 2017). In vivo administration of the P2X7R antagonist A438079 in the CD28^−/−^, IFNγ^−/−^, NOD.H‐2 h4 mouse model of salivary gland exocrinopathy ameliorated salivary gland inflammation and enhanced carbachol‐induced saliva secretion. Genetic studies have identified epistasis between P2X7R polymorphisms and HLA‐DR3 in SjD susceptibility, with the P2RX7 1405G gain‐of‐function haplotype representing a risk factor for seropositive SjD in subjects lacking HLA risk alleles (Lester et al. 2013). Elevated serum IL‐18 levels in these patients are consistent with P2X7R activation, collectively implicating P2X7R function in SjD pathogenesis.

#### 3.2.4 Muscarinic Receptor Autoantibodies

Circulating autoantibodies against muscarinic M3 receptors are detected in subsets of SjD patients, potentially interfering with parasympathetic stimulation of Ca^2+^‐dependent secretion (Bacman et al. 1996). While the functional significance of these antibodies remains incompletely understood, they may contribute to secretory dysfunction in antibody‐positive individuals by disrupting the initial receptor‐mediated signaling that triggers IP3 generation and subsequent calcium mobilization (Bacman et al. 1996).

### 3.3 Ion Channel Dysfunction in Cystic Fibrosis Salivary Glands

Cystic fibrosis (CF) patients experience increased rates of dental caries, altered oral microbiome composition, and subtle changes in saliva composition that may contribute to oral health complications (Almeslet et al. 2022). The elevated salivary electrolyte concentrations alter buffering capacity and antimicrobial properties, potentially explaining increased caries susceptibility (Almeslet et al. 2022). CF, due to mutations in the CFTR channel, primarily manifests such as pulmonary and pancreatic disease but also affects salivary gland function (Wine 1999; Quinton 2008).

#### 3.3.1 CFTR‐Dependent Ductal Dysfunction

CFTR localizes predominantly to the apical membrane of ductal epithelia in salivary glands, where it mediates Cl^−^ reabsorption from luminal fluid and facilitates HCO_3_^−^ secretion (Quinton 2008; Zinn et al. 2015; Crawford et al. 1991). The most clinically apparent manifestation of salivary CFTR dysfunction is elevated Na^+^ and Cl^−^ concentrations in the saliva of CF patients (Zinn et al. 2015; Roomans 2010). Saliva from CF patients contains 2–5 fold elevations in Na^+^ and Cl^−^ compared to healthy controls (Zinn et al. 2015; Roomans 2010). This biochemical abnormality forms the basis of the sweat chloride test, the diagnostic gold standard for CF, and analogous alterations occur in salivary chloride concentrations (Roomans 2010). CFTR potentiators provide direct evidence for the channel's functional importance in salivary secretion. Becq et al. demonstrated that subcutaneous injection of the water‐soluble CFTR potentiator MPB‐07 enhanced isoprenaline‐induced salivary secretion in a dose‐dependent manner in Cftr^+/+^ mice but not in Cftr^−/−^ mice (Noël et al. 2008). Similarly, the isoflavone genistein, another CFTR potentiator, stimulated salivary secretion only in wild‐type animals (Noël et al. 2008). These results demonstrated for the first time in vivo pharmacological stimulation of salivary secretion by CFTR potentiators and established that this chloride channel plays an important role in β‐adrenergic‐mediated salivary gland physiology.

#### 3.3.2 ENaC Co‐Regulation With CFTR

A critical finding from the Cftr^ΔF/ΔF^ mouse model was the dramatic reduction in α‐ENaC subunit expression in both the plasma membrane and total cellular protein levels of submandibular glands, while β‐ and γ‐ENaC expression remained unchanged (Catalán et al. 2010). The coordinate loss of both CFTR and ENaC function explains why CF salivary glands fail to adequately reabsorb both Na^+^ and Cl^−^ from ductal fluid, resulting in elevated salivary electrolyte concentrations. In the sweat duct, CFTR is the only apical pathway for chloride, and when Na^+^ attempts to flow out through remaining sodium‐selective pathways, it is unaccompanied by Cl^−^, creating an excess of negative charge that prevents further Na^+^ absorption (Quinton 2007). While this mechanism is less severe in salivary ducts due to alternative chloride pathways, the coordinate regulation of CFTR and ENaC appears conserved.

## 4 Ion Channels at the Frontier of Therapeutic Targeting and Diagnostics in Salivary Gland Disorders

The mechanistic understanding of ion channel dysfunction in salivary gland pathology has catalyzed the development of novel diagnostic and therapeutic strategies. Current treatment options for xerostomia remain limited to symptomatic management with saliva substitutes and muscarinic agonists, which provide only transient relief and are associated with significant side effects (Jensen et al. 2010; Turner 2016). The identification of specific ion channels as rate‐limiting steps in secretory dysfunction has enabled the development of targeted pharmacological agents, gene therapy approaches, and regenerative strategies that address underlying molecular defects rather than only improving symptoms (Table 2).

**TABLE 2:** Therapeutic approaches targeting ion channels in salivary gland disorders.

Therapeutic approach	Target	Mechanism of action	Key findings	References
Muscarinic agonists (pilocarpine, cevimeline)	M3 receptors	Activate muscarinic receptors to trigger IP_3_‐mediated Ca^2+^ release, stimulating downstream ANO1 and K^+^ channel activation for fluid secretion. Cevimeline additionally stimulates substance P release for amplified secretory response	Two‐fold increase in salivary flow vs. placebo; cevimeline produces sustained 4–6 h response with better tolerability	(Kapourani et al. 2022; Vivino et al. 1999; Rieke et al. 1995; Braga et al. 2009; Fox et al. 2001; Chambers, Jones, et al. 2007; Chambers, Posner, et al. 2007; Brimhall et al. 2013; Suzuki et al. 2013; Gil‐Montoya et al. 2016; Taniguchi et al. 2019)
ANO1/TMEM16A modulators	ANO1 channel	Activators (Eact) increase Cl^−^ conductance at zero calcium; potentiators (Fact) reduce EC_50_ for Ca^2+^‐dependent activation. Amplify physiological stimuli rather than inducing autonomous secretion	Restore salivary secretion in preclinical models; offer CFTR‐independent pathway for CF therapy	(Turner 2016; Ramsey et al. 2011)
CFTR modulators (ivacaftor, elexacaftor/tezacaftor)	CFTR channel	Potentiators increase channel open probability; correctors improve trafficking of mutant CFTR (e.g., F508del) to plasma membrane, restoring ductal Cl^−^/HCO_3_^−^ transport	Normalize sweat chloride in responsive patients; potential to reduce salivary electrolyte abnormalities and caries risk in CF	(Heijerman et al. 2019; LaRusch et al. 2015; Angyal et al. 2021)
AAV2‐hAQP1 gene therapy	AQP1	AAV2‐mediated AQP1 expression in radiation‐surviving ductal cells enables transcellular water flux from interstitium into ductal lumen, bypassing damaged acinar secretory machinery	Phase 1 AQUAx trial: 67% response rate; 112% improvement in unstimulated salivary flow; FDA RMAT designation granted December 2024; Phase 2 trial (NCT05926765) ongoing	(Baum et al. 2012; Shan et al. 2005; Alevizos et al. 2017; Corden et al. 2017; Lai et al. 2016; Brennan et al. 2025)
Ultrasound‐assisted gene transfer (UAGT)	AQP1	Non‐viral plasmid delivery using clinical ultrasound and microbubbles to transiently permeabilize cell membranes	Bilateral salivary flow restoration to pre‐injury levels in irradiated minipigs; no inflammation or morphological alterations	(Wang et al. 2015; Trombetta et al. 2015)
Organoid/tissue engineering	Functional tissue replacement	3D organoids derived from SGS/PCs express functional ion channels (ANO1, AQP5, NKCC1, M3R) and respond to cholinergic stimulation with Ca^2+^ mobilization. Scaffolds provide ECM‐mimetic architecture	FGF10‐induced organoids develop into mature secretory tissue with branching morphology; decellularized ECM supports functional ion channel expression	(Lombaert et al. 2017; Langthasa et al. 2025; Matsuno et al. 2025; Sui et al. 2020; Tanaka et al. 2018; Hajiabbas et al. 2022)

### 4.1 Diagnostic Potential of Ion Channel Expression and Function

Ion channel expression patterns and functional alterations act as powerful biomarkers for salivary gland pathology. The AQP5 redistribution characteristic of SjD (Section 3.2.1) also holds diagnostic value. Quantitative computer‐assisted microscopy reveals that Sjögren's patients exhibit significantly higher basolateral AQP5 labeling indices and correspondingly lower apical labeling compared to controls, with this pattern being specific to SjD rather than other causes of xerostomia such as sarcoidosis or nonspecific sialoadenitis (Konttinen et al. 2005). Moreover, the degree of AQP5 redistribution correlates directly with salivary flow deficits and is independent of the focus score, supporting its utility as an early pathogenic and diagnostic marker (Chivasso et al. 2023; Yoshimura et al. 2016).

Autoantibodies against ion channels provide additional diagnostic value. Anti‐AQP5 autoantibodies can be detected in serum by ELISA, a study reporting a diagnostic area under the curve of 0.86, sensitivity of 95%, and specificity of 70% for distinguishing primary SjD from other connective tissue diseases (Wang et al. 2024). Interestingly, anti‐AQP5 levels did not correlate with traditional disease activity markers (ESULAR index, anti‐SSA/SSB titers, or salivary flow rates), suggesting these antibodies may represent an independent diagnostic pathway (Wang et al. 2024). The presence of anti‐M3 muscarinic receptor autoantibodies in a subset of Sjögren's patients further implicates receptor‐mediated dysfunction in pathogenesis, though their diagnostic sensitivity remains lower than traditional markers (Bacman et al. 1996).

Functional imaging exploits ion channel‐dependent processes. Salivary gland scintigraphy using technetium‐99 m pertechnetate reveals that in radiation‐damaged glands, radioisotope uptake (reflecting NKCC1‐mediated trapping) remains relatively intact while secretory excretion (dependent on apical ANO1 and AQP5) is severely impaired, providing functional evidence that secretory ion channel dysfunction precedes glandular destruction (Nagler 2002). This dissociation between uptake and excretion phases offers prognostic information about residual gland function and may guide therapeutic decisions regarding secretagogue trials versus gene therapy approaches.

### 4.2 Pharmacological Modulation of Ion Channel Activity

Pharmacological targeting of ion channels provides the foundation of current xerostomia management, with muscarinic agonists representing the only FDA‐approved disease‐modifying therapies. Beyond these established agents, emerging small‐molecule modulators of ANO1 and CFTR offer mechanistic precision by directly enhancing chloride conductance independent of upstream signaling. These pharmacological approaches are most effective when functional secretory machinery remains partially intact, distinguishing them from gene therapy and regenerative strategies designed to restore absent or severely damaged channels.

#### 4.2.1 Muscarinic Receptor Agonists

Pilocarpine and cevimeline represent the only FDA‐approved pharmacological therapies specifically targeting salivary ion channels for xerostomia management. Pilocarpine, a nonselective muscarinic agonist, activates all five muscarinic receptor subtypes (M1‐M5) with therapeutic effects mediated primarily through M3 receptors on acinar cells (Kapourani et al. 2022; Barbe 2018). The standard dose of 5 mg administered orally three to four times daily produces muscarinic effects within 20 min and has an elimination half‐life of 0.76–1.3 h (Kapourani et al. 2022). Multiple randomized, placebo‐controlled trials have demonstrated pilocarpine's efficacy in increasing resting and stimulated salivary flow and improving subjective oral dryness in both SjD and post‐radiation xerostomia, with a two‐fold increase in salivary flow rates compared to placebo (Kapourani et al. 2022; Vivino et al. 1999; Rieke et al. 1995).

Cevimeline exhibits greater selectivity for M1 and M3 receptors, conferring theoretical advantages in side effect profile (Braga et al. 2009; Fox et al. 2001). At the standard dose of 30 mg three times daily, cevimeline produces moderate and sustained increases in salivary flow lasting 4–6 h, exceeding pilocarpine's duration of action due to a longer elimination half‐life (Braga et al. 2009; Chambers, Jones, et al. 2007; Chambers, Posner, et al. 2007). Head‐to‐head comparisons reveal no significant difference in efficacy between pilocarpine and cevimeline, though discontinuation rates favor cevimeline due to better tolerability during long‐term administration (Brimhall et al. 2013; Noaiseh et al. 2014). Mechanistically, cevimeline specifically stimulates substance P release in salivary glands via M3 receptor activation, providing neuropeptide‐mediated amplification of secretory responses (Suzuki et al. 2013).

Adverse effects common to both therapies arise from systemic muscarinic receptor stimulation and include sweating as the predominant symptom, along with nausea, diarrhea, urinary frequency, and rhinitis (Kapourani et al. 2022; Braga et al. 2009). These cholinergic side effects necessitate caution in patients with cardiovascular disease, asthma, narrow‐angle glaucoma, or those taking medications that may interact with cholinergic pathways (Gil‐Montoya et al. 2016). Continuous administration of pilocarpine upregulates M3 receptor expression in salivary glands, potentially enhancing therapeutic efficacy over time, though this adaptation may also contribute to tolerance in some patients (Taniguchi et al. 2019).

#### 4.2.2 ANO1/TMEM16A Modulators

Small‐molecule modulators of ANO1 represent a promising frontier in xerostomia pharmacotherapy. High‐throughput screening has identified two classes of ANO1‐selective compounds: activators (Eact) that increase chloride conductance even at zero calcium, and potentiators (Fact) that reduce the EC_50_ for calcium‐dependent activation (Namkung, Yao, et al. 2011; Oh and Jung 2016). The most potent activator compounds produce chloride currents with EC_50_ values of 3–6 μM, comparable in magnitude to currents induced transiently by calcium‐elevating purinergic agonists but with more sustained kinetics (Namkung, Yao, et al. 2011; Oh and Jung 2016).

ANO1 activators offer mechanistic advantages over nonselective calcium‐mobilizing agonists like pilocarpine. Since chloride secretion requires coordinated activation of both ANO1 and basolateral potassium channels, TMEM16A‐targeted activators would primarily amplify physiological stimuli rather than induce secretion autonomously, potentially reducing side effects (Namkung, Yao, et al. 2011). Preclinical studies demonstrate that ANO1 activators restore salivary secretion and intestinal motility in tissue preparations, suggesting broad therapeutic potential (Romanenko et al. 2010; Namkung, Yao, et al. 2011).

ANO1 inhibitors have been extensively characterized for other indications. T16Ainh‐A01, an aminophenylthiazole with IC_50_ ~ 1 μM, fully blocks ANO1 currents in salivary gland cells, providing pharmacological confirmation that ANO1 carries nearly all calcium‐activated chloride current in this tissue (unlike airways and intestine where ANO1 contributes only partially) (Yang et al. 2008; Namkung, Phuan, and Verkman 2011). This tissue‐specific dominance positions ANO1 as an exceptionally specific target in salivary glands, though inhibitors would obviously be contraindicated for xerostomia treatment.

#### 4.2.3 Aquaporin Modulators

Pharmacological upregulation of AQP expression has been explored through multiple mechanisms. Chemical demethylation studies demonstrate that the AQP1 promoter is silenced by methylation in salivary ductal cells, and global demethylation induces robust AQP1 expression comparable to adenoviral transduction (Wang et al. 2017). This finding has driven interest in epigenetic editing approaches using CRISPR‐dCas9‐based synthetic transcriptional activator systems, which successfully activate native AQP1 expression without introducing exogenous DNA (Wang et al. 2017).

#### 4.2.4 CFTR Modulator Therapies

Small‐molecule CFTR modulators, including potentiators (ivacaftor) that increase channel open probability and correctors (lumacaftor, tezacaftor, elexacaftor) that improve trafficking of mutant CFTR to the plasma membrane, have revolutionized CF treatment (Ramsey et al. 2011; Heijerman et al. 2019). While primarily developed for pulmonary and pancreatic manifestations, these therapies normalize sweat chloride concentrations in responsive patients, demonstrating functional CFTR correction in exocrine glands (Ramsey et al. 2011; Heijerman et al. 2019). Whether CFTR modulators improve salivary gland function or reduce oral complications in CF remains incompletely studied. Given that salivary dysfunction is relatively mild in CF and alternative secretory pathways exist, dramatic improvements may not be observed. However, normalization of salivary electrolyte composition could potentially reduce caries risk and improve oral health outcomes (Heijerman et al. 2019). The insights gained from studying CFTR in salivary glands inform broader understanding of epithelial ion transport and have potential relevance for other CFTR‐related disorders including pancreatitis, where partial CFTR dysfunction contributes to disease pathogenesis (LaRusch et al. 2015; Angyal et al. 2021).

### 4.3 Gene Therapy Approaches Targeting Ion Channels

Gene therapy approaches offer permanent or sustained restoration of ion channel expression in radiation‐damaged or genetically deficient salivary tissue, addressing the fundamental limitation of pharmacological strategies that require residual functional machinery. Viral vector‐mediated delivery of aquaporin‐1 has advanced furthest toward clinical implementation, demonstrating that expression of water channels in surviving ductal cells can bypass acinar destruction to restore fluid secretion. The progression from first‐generation adenoviral vectors through optimized AAV serotypes to non‐viral ultrasound‐assisted delivery illustrates the field's evolution toward safer, more durable, and potentially repeatable therapeutic gene transfer.

#### 4.3.1 Adenoviral AQP1 Gene Transfer

The initial proof of this approach came from pioneering work demonstrating that AQP1 expression in ductal cells enables transcellular fluid flux from interstitial compartments into the ductal lumen, effectively bypassing destroyed acinar tissue (Baum et al. 2009, 2012). A Phase I clinical trial (NCT00372320) using first‐generation serotype 5 adenoviral vector (AdhAQP1) in patients with radiation‐induced xerostomia established both safety and therapeutic efficacy (Baum et al. 2012; Shan et al. 2005). Eleven patients received AdhAQP1 delivered to a single parotid gland via retrograde ductal cannulation at doses ranging from 4.8 × 10^7^ to 5.8 × 10^10^ viral particles. Results demonstrated dose‐dependent increases in parotid salivary flow to approximately 35%–80% of pre‐irradiation levels on day 3 post‐treatment, with responders showing sustained improvements in both objective flow measurements (71%–500% above baseline) and subjective xerostomia symptoms for 2–3 years (Baum et al. 2012; Shan et al. 2005). Remarkably, long‐term follow‐up (3–4.7 years) revealed persistent elevation in salivary flow despite the known transient expression typical of first‐generation adenoviral vectors (Alevizos et al. 2017). Core needle biopsies from responders 3.1 years post‐treatment confirmed AQP1 protein expression in both luminal and basolateral membranes of transduced acini, providing direct evidence for unexpectedly durable transgene expression (Alevizos et al. 2017). The clinical trial revealed no serious adverse events, minimal changes in hematology or clinical chemistry parameters, and limited vector biodistribution (AdhAQP1 detected only transiently in saliva and blood, with sporadic low‐level detection in sublingual glands sharing common ducts) (Shan et al. 2005; Baum et al. 2009). However, periductal inflammation observed histologically in some specimens suggested immune‐mediated clearance of transduced cells or vector in a subset of patients, likely accounting for non‐responders.

#### 4.3.2 Adeno‐Associated Viral Vectors

To address adenovirus‐associated immunogenicity and enhance duration of transgene expression, a second‐generation clinical trial (NCT02446249) using adeno‐associated virus serotype 2 (AAV2‐hAQP1) was initiated in 2016 (Corden et al. 2017). AAV2 offers theoretical advantages including reduced immunogenicity, episomal persistence enabling longer expression, and a superior safety profile (Nakamura et al. 2022). Preclinical studies in minipigs demonstrated that AAV2‐AQP1 delivery restored salivary flow comparably to adenoviral vectors without causing periductal inflammation (Nakamura et al. 2022). Remarkably, LAMP3‐overexpressing conditions (as occur in SjD) paradoxically enhance AAV2 uptake via promoted endolysosomal pathways, potentially improving transduction efficiency in autoimmune contexts (Lai et al. 2016). The Phase 1 AQUAx trial (NCT04043104) has now been completed with results demonstrating both safety and efficacy of AAV2‐hAQP1 for grade 2/3 radiation‐induced xerostomia (Brennan et al. 2025). In this multicenter, open‐label, dose‐escalation study, 24 participants received AAV2‐hAQP1 delivered to one or both parotid glands via Stensen duct cannulation at doses ranging from 1 × 10^11^ to 3 × 10^12^ vg/gland (unilateral) or 3 × 10^10^ to 1 × 10^12^ vg/gland (bilateral). No treatment‐related serious adverse events or dose‐limiting toxicities were observed throughout the 12‐month follow‐up period. Statistically significant improvements in patient‐reported outcome instruments were observed by day 30 and maintained throughout month 12. The mean percent change from baseline was −39.5% for the Xerostomia‐specific Questionnaire (XQ) Total Score and −42.2% for the MD Anderson Symptom Inventory‐Head and Neck (MDASI‐HN) Dry Mouth question, with the mean Global Rate of Change Questionnaire (GRCQ) symptom score reaching 3.8 at month 12 (Brennan et al. 2025). Overall, 16 of 24 participants (67%) reported clinically meaningful improvement of ≥ 8 points in XQ Total Score, and 19 of 24 (79%) reported important improvements based on the GRCQ. Objective measurements demonstrated a mean 112.5% improvement in unstimulated whole saliva flow rate at month 12, with a trend toward improved stimulated flow (Brennan et al. 2025).

Based on these promising clinical data, the U.S. Food and Drug Administration granted Regenerative Medicine Advanced Therapy (RMAT) designation to AAV2‐hAQP1 in December 2024, recognizing preliminary clinical evidence of potential benefit in this condition representing a completely unmet medical need. Grade 2/3 radiation‐induced xerostomia affects approximately 30%–40% of patients treated with radiation for head and neck cancer, with an estimated 170,000 current patients in the United States and approximately 15,000 new patients annually. A Phase 2 randomized, double‐blind, placebo‐controlled study (AQUAx2; NCT05926765) is currently enrolling participants at multiple sites in the United States, Canada, and the United Kingdom, with regulatory alignment achieved for this trial to potentially serve as a pivotal study in support of a future Biologics License Application.

### 4.4 Organoid Technology

Three‐dimensional organoid culture systems have emerged as powerful platforms for modeling salivary gland development, disease pathogenesis, and regenerative potential (Lombaert et al. 2017; Langthasa et al. 2025; Matsuno et al. 2025). Human submandibular gland stem/progenitor cells (hSMGepiS/PCs) can be induced to form salivary gland organoids through FGF10 treatment, promoting expression of differentiation markers, structural organization, and responsiveness to neurotransmitters (including calcium mobilization upon cholinergic stimulation, indicating functional ion channel assembly) (Sui et al. 2020). When these FGF10‐induced organoids were combined with embryonic day 12.5 mouse salivary gland mesenchyme and transplanted into renal capsules, they developed into structurally mature and functional salivary tissue with branching morphology, demonstrating that human SGS/PCs respond productively to developmental niche signals (Sui et al. 2020). Significantly, organoid‐derived tissues express appropriate ion channels and transporters essential for secretory function, including ANO1, AQP5, NKCC1, and M3 receptors, validated through immunofluorescence and functional calcium release assays (Sui et al. 2020). Recent advances include magnetic 3D bioassembly techniques combining salivary organoids with human dental pulp stem cells, which when transplanted into irradiated mouse glands, promoted both epithelial and neuronal regeneration (Tanaka et al. 2018).

## 5 Conclusion

The conjunction of mechanistic insight and therapeutic innovation has fundamentally repositioned ion channels from passive channels of epithelial transport to actionable therapeutic targets. Restoration of water permeability through AAV2‐hAQP1 gene therapy has demonstrated sustained improvement in salivary flow and earned regulatory recognition, establishing proof‐of‐concept that molecular correction of secretory pathways can reverse glandular hypofunction. Pharmacological modulation of chloride and calcium channels, along with targeted interference in inflammatory ion channel signaling, further expands the therapeutic repertoire beyond symptomatic management. Meanwhile, regenerative and organoid‐based approaches increasingly rely on ion channel expression and calcium signaling competence as functional benchmarks of true secretory restoration.

Future progress will depend on stratifying patients according to specific ion channel dysfunction profiles and integrating pharmacologic, genetic, and regenerative strategies to address distinct pathogenic mechanisms. By positioning ion channels at the center of both disease pathogenesis and therapeutic repair, this framework supports the development of precision approaches capable of transforming xerostomia management across radiation‐induced, autoimmune, and inherited etiologies.

## Author Contributions

T.M.A.E.‐A., S.N.V., V.D., P.A., M.I.S, A.A.‐S., and B.B.S. wrote and edited the manuscript. All authors have read and agreed to the published version of the manuscript.

## Funding

This work was supported by grants from the National Institutes of Health (NIDCR) (Grants R01DE017102; R01DE031716; R01DE032317).

## Conflicts of Interest

The authors declare no conflicts of interest.

## Data Availability

The data that support the findings of this study are available from the corresponding author upon reasonable request.
